# Modeling the transmission and control of Zika in Brazil

**DOI:** 10.1038/s41598-017-07264-y

**Published:** 2017-08-10

**Authors:** Liping Wang, Hongyong Zhao, Sergio Muniz Oliva, Huaiping Zhu

**Affiliations:** 1 Department of Mathematics, Nanjing University of Aeronautics and Astronautics, Nanjing, 210016 P.R. China; 2 Departamento de Matemática Aplicada, Instituto de Matemática e Estatística, Universidade de São Paulo, Rua do Matão, 1010, Cidade Universitária, CEP 05508-090 São Paulo, SP Brazil; 3 Lamps and Department of Mathematics and Statistics, York University, Toronto, ON M3J 1P3 Canada

## Abstract

Zika virus, a reemerging mosquito-borne flavivirus, started spread across Central and Southern America and more recently to North America. The most serious impacted country is Brazil. Based on the transmission mechanism of the virus and assessment of the limited data on the reported suspected cases, we establish a dynamical model which allows us to estimate the basic reproduction number *R*_0_ = 2.5020. The wild spreading of the virus make it a great challenge to public health to control and prevention of the virus. We formulate two control models to study the impact of releasing transgenosis mosquitoes (introducing bacterium Wolbachia into *Aedes aegypti*) on the transmission of Zika virus in Brazil. Our models and analysis suggest that simultaneously releasing Wolbachia-harboring female and male mosquitoes will achieve the target of population replacement, while releasing only Wolbachia-harboring male mosquitoes will suppress or even eradicate wild mosquitoes eventually. We conclude that only releasing male Wolbachia mosquitoes is a better strategy for control the spreading of Zika virus in Brazil.

## Introduction

Zika virus is a reemerging mosquito-borne flavivirus very much similar to dengue. It is transmitted through the bite of an infected mosquito of the genus *Aedes*. Zika virus has other possible routes of transmission including mother to child, sexual and blood transfusion^1–3^. Symptoms of Zika infection are generally mild and self-limiting, and infected individual may experience rash, fever, pain and headache due to the flavivirus. Usually, these symptoms resolve in about a week without medical treatment^4^. Nevertheless, there is now scientific consensus that Zika virus is a cause of microcephaly and Guillain-Barr*é* syndrome^5^, and links to other neurological complications are also being investigated.

**Figure 1:**
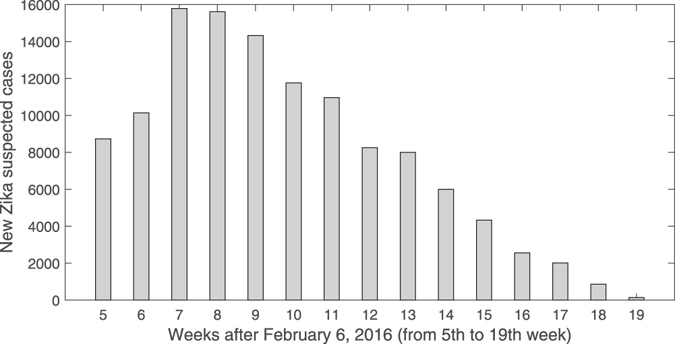
Reported suspected Zika cases in Brazil from February 6 to June 2, 2016.

Zika was first isolated from rhesus monkeys in Zika forest, Uganda in 1947^6^. Few human cases were reported until the first known outbreak in Yap Island, Micronesia, during April-July 2007^7^. Later on, an outbreak occurred in French Polynesia between October 2013 and April 2014^8^. Since then, Zika virus is no longer a mild infection limited to Africa and Asia any more^9–11^, it has spread rapidly across continents, swept over from central and southern America and arrived in North America in 2016. On February 1, 2016, WHO declared Zika as a “Public Health Emergency of International Concern”^12^. Up to June 2, 2016, 363,990 suspected and 52,003 confirmed cumulative Zika cases were reported by countries and territories in the Americas, in which 42% suspected and 77% confirmed cumulative cases were notified for Brazil (see Supplementary Fig. S1). Brazil had experienced a large Zika epidemic in 2015 and 2016. The first reported Zika case in Brazil was from March 2015 in the state of Bahia, Northeast Brazil^13^. In 2016, as shown in Fig. 1, the new reported suspected cases reached a peak of 15,784 in the week of Feb. 20 (7th week of the year).

Currently, there are no antiviral therapies to treat the infection, and there has been great effort to develop vaccines for Zika virus. Controlling of vector mosquitoes remains the most effective measure for prevention of Zika and other mosquito-borne diseases^14, 15^. However, the traditional use of insecticides is often excessively expensive and environmental undesirable, moreover, it leads to insecticide resistance^15^. Therefore, it is very important to search for novel technologies to control the spreading of Zika and other mosquito-borne infections.

Wolbachia bacterium as an innovative approach for controlling dengue fever was introduced into *Aedes aegypti* mosquitoes^16–20^. In order to block the spread of dengue fever, mosquitoes implanted with different strains of Wolbachia bacteria were released in a few countries. The first releases were those infected with *wMel* Wolbachia (strong anti-dengue properties and low fitness costs) in Yorkeys Knob and Gordonvale in north-eastern Australia in 2011^19^. Later, Wolbachia mosquitoes was released in September 2014 in Tubiacanga, north of Rio de Janeiro, to block the spread of dengue^21^.

Currently, there is no report on releasing transgenosis mosquitoes to control Zika. Yet, Dutra *et al*.^17^ reported that *Aedes aegypti* harboring Wolbachia was highly resistant to infection with two currently circulating Zika virus isolates from the recent Brazilian epidemic. The Wolbachia bacteria lives within testes and ovaries of their hosts and is passed from one generation to the next through the eggs of mosquitoes. Thus, they can interfere with the reproductive process of mosquitoes, causing phenomena such as cytoplasmic incompatibility (CI), parthenogenesis and feminization of genetic males. Appearances of these phenotypes depend on the host species and Wolbachia types. CI causes uninfected Wolbachia females that mate with infected Wolbachia males to rarely produce fertile eggs, while infected Wolbachia females are not affected. This gives infected Wolbachia females an advantage and helps the bacteria to spread quickly through the mosquito population^22–25^, which effectively block the reproduction of the vector mosquitoes. Therefore CI leads to complex dynamics for the mosquito population in the presence of Wolbachia, and also affects the transmission dynamics of Zika.

There have been some mathematical modeling studies to investigate the role of Wolbachia-harboring mosquitoes in control of dengue fever^26, 27^. Zhang *et al*.^26, 27^ established and studied birth pulse models of Wolbachia-induced CI by considering density dependent death rate in controlling of dengue fever. Several models had been developed to explore the effect on controling Zika virus^28–32^. For example, Kucharski *et al*.^28^ employed a mathematical model to examine the 2013–14 outbreak on the six major archipelagos of French Polynesia and predicted that it would take 12–20 years before there are sufficient number of susceptible individuals for Zika to re-emerge and become autochthonous endemic. Sexual transmission is not considered in these models, but it is indeed an important route of spreading of Zika virus. Gao *et al*.^30^ studied a model with sexual transmission and concluded that sexual transmission increases the risk of infection and epidemic size and prolongs the outbreak.

The control models of the emerging Zika virus, by contrast to dengue fever, have not been well-studied. In particular, few mathematical models are developed to explore the effect of Wolbachia on the transmission and spread of Zika virus. In this paper, we will first establish a model without including control measures and use the model to simulate the reported suspected human cases of Zika in Brazil for the period from February 6 to June 2, 2016. This model will allow us to estimate the basic reproduction number. What is more, we will extend the model to incorporate the effect of releasing Wolbachia-harboring mosquitoes on control of the transmission of Zika. By comparing different strategies of releasing Wolbachia-harboring mosquitoes, we will conclude that only releasing male Wolbachia mosquitoes will be a more effective option to control the spread of Zika virus.

## Materials and Methods

### Data

There is no systematic data of human infection available to allow comprehensive modeling studies of the transmission of Zika in Brazil. What is available to us is the time series of weekly Zika cases reported by the World Health Organization^33^ and the Brazil Ministry of Health^34^. The data contains suspected cases, confirmed cases, accumulated cases and cases of death due to Zika infection. Patients are classified according to the WHO case definition^35^. We choose the fifth week as the starting date of the first observation and use the weekly reported suspected cases of Zika in Brazil from February 6 to June 2, 2016 for our study.

### A dynamical model without control measures

**Figure 2:**
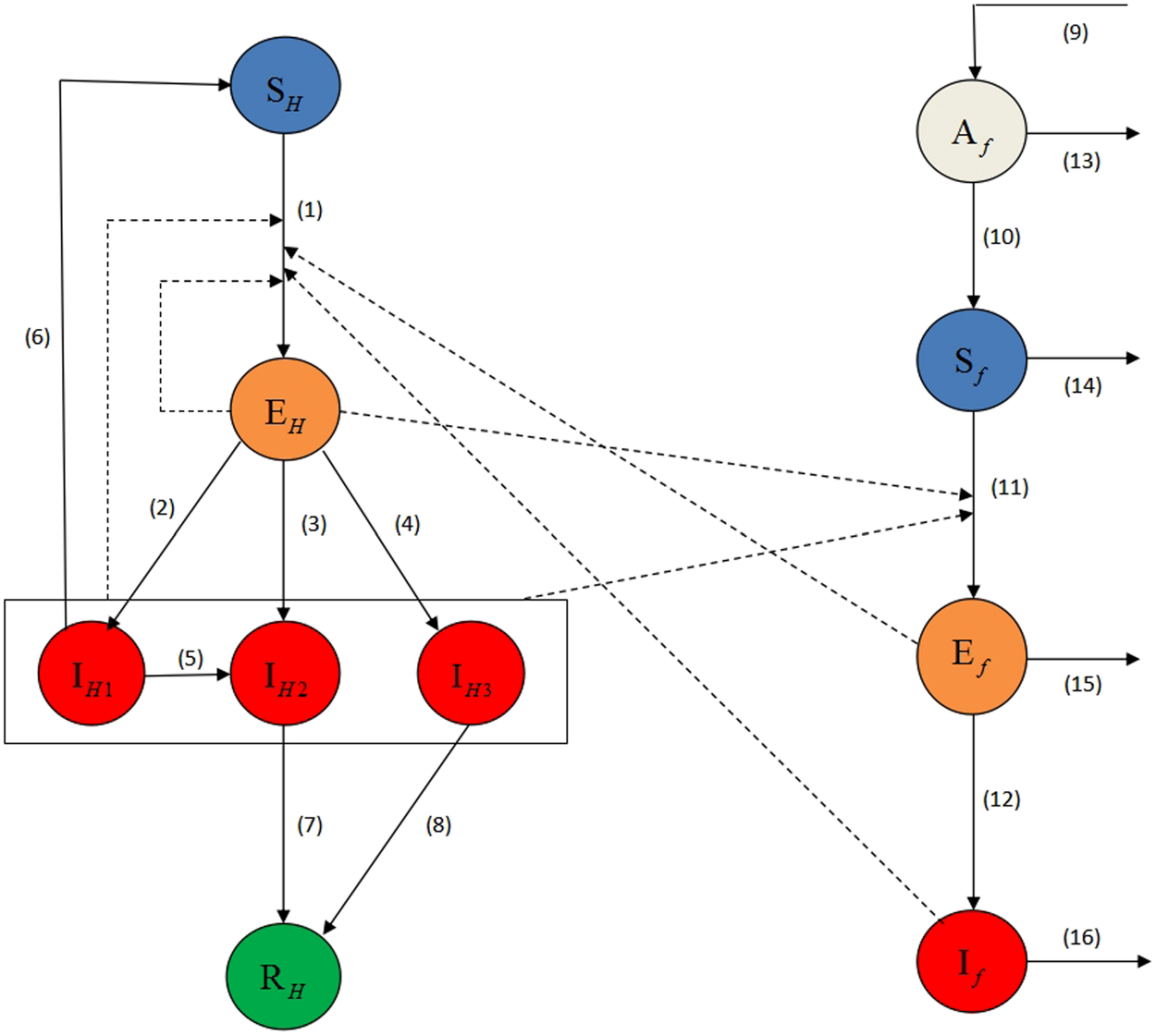
Dynamical transmission flow diagram of Zika among humans and mosquitoes. Subscripts *H* and *f* indicate humans and Wolbachia-free mosquitoes, and *A*, *S*, *E*, *I*, and *R* indicate aquatic, susceptible, exposed, infectious and recovered populations, respectively. The parameters are given in Table 1.

We simulate the transmission dynamics of Zika in Brazil by using a compartmental model^51, 52^ an approach has been widely used in the study of spreading of infectious diseases. As shown in Fig. 2, in the flow diagram of the transmission, human population are modeled using a susceptible-exposed-infectious-removed (SEIR) framework. Mosquitoes are classified in the classes of susceptible, exposed and infectious (SEI) respectively. Total human population at time *t*, denoted by *N*_*H*_(*t*), is sub-divided into four categories: susceptible humans *S*_*H*_(*t*), exposed humans *E*_*H*_(*t*), infectious humans *I*_*H*_(*t*) and recovered humans *R*_*H*_(*t*). The infectious humans *I*_*H*_(*t*) are divided into three classes: suspected cases *I*_*H*1_(*t*), confirmed cases *I*_*H*2_(*t*) and asymptomatic cases *I*_*H*3_(*t*). So *N*_*H*_ = *S*_*H*_ + *E*_*H*_ + *I*_*H*1_ + *I*_*H*2_ + *I*_*H*3_ + *R*_*H*_. Since only female mosquitoes bite to take blood meals, male mosquitoes are ignored in this model. We denote the total number of mosquitoes at time *t* as *N*_*f*_(*t*) which includes adult female mosquitoes *F*_*f*_ and total mosquitoes in aquatic stage *A*_*f*_(*t*). Here we combine the egg, larval and pupal stages as one aquatic stage, hence *N*_*f*_ = *A*_*f*_ + *F*_*f*_. For adult female mosquitoes *F*_*f*_, we will classify them into susceptible *S*_*f*_(*t*), exposed *E*_*f*_(*t*) and infectious *I*_*f*_(*t*), then *F*_*f*_ = *S*_*f*_ + *E*_*f*_ + *I*_*f*_. We denote *M*_*f*_ as the total number of adult male mosquitoes.

**Table 1:** Parameter descriptions, values and sources.

Parameter	Interpretation	Range	Value	Source
*a*	Biting rate of Wolbachia-free mosquitoes (Week^−1^)	(2.1, 7)	2.8	36
*a*_1_	Biting rate of Wolbachia-harboring mosquitoes (Week^−1^)		0.95 *a*	37
*m*	Average ratio of mosquitoes to humans (mosquitoes per human)	(1, 10)	2.3	38
*β*_1_	Transmission probability from	(0.1, 0.75)	0.6	36
	Wolbachia-free mosquitoes to humans (Dimensionless)			
*β*_11_	Transmission probability from		0.5 *β*_1_	39
	Wolbachia-harboring mosquitoes to humans (Dimensionless)			
*β*_2_	Transmission probability from	(0.3, 0.75)	0.4	40
	humans to Wolbachia-free mosquitoes (Dimensionless)			
*β*_22_	Transmission probability from		0.5 *β*_2_	39
	humans to Wolbachia-harboring mosquitoes (Dimensionless)			
*β*_3_	Sexual transmission rate from humans to humans (Dimensionless)	(0, 0.1)	0.04	Estimated
*p*	Misdiagnosed proportion in the suspected cases (Dimensionless)	(0, 1)	0.122	Estimated
*z*	Proportion of *E*_*H*_ enter the *I*_*H*2_ compartment (Dimensionless)	(0, 1)	0.4071	Estimated
*ϕ*_*H*_	Proportion of susceptible humans who took effective precautions (Dimensionless)	(0, 1)	0.31	Estimated
*l*	The proportion of female in adult mosquitoes (Dimensionless)	(0, 1)	0.3	Estimated
*q*	The report rate of Zika new cases (Dimensionless)	(0, 1)	0.012	30
*θ*	Proportion of symptomatic infection (Dimensionless)	(0.1, 0.27)	0.13	8
1/*γ*_1_	The mean time of suspected cases (Week)	(3/7, 1)	4/7	41
1/*γ*_2_	Time from confirmed cases enter the recovery (Week)	(13/7, 30/7)	2	43, 44
1/*γ*_3_	Time from asymptomatic cases enter the recovery (Week)	(5/7, 15/7)	6/7	Assumed
*σ*_1_	The transition rate from exposed cases	(0, 1)	1/4	Assumed
	to the suspected and asymptomatic cases (Week^−1^)			
*σ*_2_	The transition rate from exposed cases to the confirmed cases (Week^−1^)	(0, 1)	1/5	Assumed
*ρ*_*f*_	Reproductive rate of Wolbachia-free mosquitoes (Week^−1^)	(7, 17.5)	7.07	26
*ρ*_*h*_	Reproductive rate of Wolbachia-harboring mosquitoes (Week^−1^)		0.95 *ρ*_*f*_	45
*ω*_*f*_	Maturation rate of aquatic Wolbachia-free mosquitoes (Week^−1^)	(7/17, 7/6)	7/10	46
*ω*_*h*_	Maturation rate of aquatic Wolbachia-harboring mosquitoes (Week^−1^)	(7/17, 7/6)	7/10	46
*μ*_*fA*_	Death rate of aquatic Wolbachia-free mosquitoes (Week^−1^)	(7/20, 1)	7/8	46
*μ*_*hA*_	Death rate of aquatic Wolbachia-harboring mosquitoes (Week^−1^)	(7/20, 1)	7/8	46
*μ*_*f*_	Death rate of adult Wolbachia-free mosquitoes (Week^−1^)	(7/30, 9/10)	1/3	46
*μ*_*h*_	Death rate of aduit Wolbachia-harboring mosquitoes (Week^−1^)		1.1 *μ*_*f*_	45, 47
1/*σ*_*f*_	Progression from exposed to infectious of Wolbachia-free mosquitoes (Week)	(8/7, 12/7)	10/7	13, 42
1/*σ*_*h*_	Progression from exposed to infectious of Wolbachia-harboring mosquitoes (Week)	(8/7, 12/7)	10/7	13, 42
*k*_1_, *k*_2_, *k*_3_	Modification parameter	(0, 1)	0.1	48–50
*η*_1_, *η*_2_, *η*_3_, *η*_*h*_	Modification parameter	(0, 1)	0.01	48–50
*η*_*f*_	Modification parameter	(0, 1)	0.4	48–50

Transmission rate of the Fig. 2

**Table:** 

Number	Transition rate
(1)	[*β*_*fH*_(1 − *ϕ*_*H*_) + *β*_*HH*_]*S*_*H*_
(2)	*θ*(1 − *z*)*σ*_1_ *E*_*H*_
(3)	*zσ*_2_ *E*_*H*_
(4)	(1 − *θ*)(1 − *z*)*σ*_1_ *E*_*H*_
(5)	(1 − *p*)*γ*_1_ *I*_*H*1_
(6)	*pγ*_1_ *I*_*H*1_
(7)	*γ*_2_ *I*_*H*2_
(8)	*γ*_3_ *I*_*H*3_
(9)	*ρ*_*f*_ *F*_*f*_(1 − (*A*_*f*_)/(*K*))
(10)	*lω*_*f*_ *A*_*f*_
(11)	*β*_*Hf*_ *S*_*f*_
(12)	*σ*_*f*_ *E*_*f*_
(13)	*μ*_*fA*_ *A*_*f*_
(14)	*μ*_*f*_ *S*_*f*_
(15)	*μ*_*f*_ *E*_*f*_
(16)	*μfIf*

as following:

In this study, we will only consider one Zika strain in the course of a outbreak. Since we will consider a case of a single outbreak of the virus in one mosquito season (within a year), human demographics will be ignored in the model. We consider *Aedes aegypti* as the only vector responsible for the transmission of Zika virus, and if we also assume that there is no control on the vector mosquitoes, then we present the transmission flow in Fig. 2.

A susceptible human may become infected with the Zika virus via the bite of an exposed or infectious mosquito during probing and feeding at a rate *aβ*_1_(*η*_*f*_ *E*_*f*_  + *I*_*f*_)/*N*_*H*_ and through sexual transmission at a rate *β*_3_(*k*_1_ *E*_*H*_ + *k*_*2*_ *I*_*H*__1_ + *I*_*H*__2_ *+ k*_3_ *I*_*H*__3_)/*N*_*H*_. In a few days, some of the exposed become infected with symptoms while the rest either remain as susceptible or become infectious yet asymptomatic. The modification parameter 0 < *η*_*f*_ < 1 accounts for the assumed reduction in transmissibility of exposed mosquitoes relative to infectious mosquitoes. It is worth emphasizing that, unlike many of the published modeling studies on Zika transmission dynamics, the current study assumes that exposed vectors can transmit Zika disease to humans (that is, 0 < *η*_*f*_). *k*_1_, *k*_2_ and *k*_3_ are the modification parameters and 0 ﻿<* k*_1﻿_, *k*_2_, *k*_3_ <1.

The aquatic mosquito population increases as the adult mosquitoes mate and breed by a rate *ρ*_*f*_ *F*_*f*_(1 *− A*_*f*_/*K*), which is limited by *K* in the logistic term to reflect the number and size of available breeding sites. It depends on the number of available hosts, including humans. We assume that *K* ∝ *N*_*H*_, and hence *K* = *mN*_*H*_. The aquatic population at a rate of *ω*_*f*_ matures into susceptible mosquitoes, in which the proportion of female is *l*. So we have always *F*_*f*_  = ( l/(1 −*l*))*M*_*f*_. Susceptible mosquitoes move to the exposed class after biting exposed or infectious humans at a rate of *β*_*Hf*_. Then they become infectious at a rate of *σ*_*f*_ *E*_*f*_ and later a death rate of *μ*_*f*_ *I*_*f*_. The transmission flow diagram is given in Fig. 2.

The per-capita contact transmission rate from natural (Wolbachia-free) mosquitoes to humans can be written as

1 $${\beta }_{fH}=a{\beta }_{1}\frac{({\eta }_{f}{E}_{f}+{I}_{f})}{{N}_{H}}=a{\beta }_{1}m\frac{({\eta }_{f}{E}_{f}+{I}_{f})}{K},$$

the per-capita infection rate from humans to Wolbachia-free mosquitoes can be modeled as

2 $${\beta }_{Hf}=a{\beta }_{2}\frac{({\eta }_{1}{E}_{H}+{\eta }_{2}{I}_{H1}+{I}_{H2}+{\eta }_{3}{I}_{H3})}{{N}_{H}},$$

the contact transmission rate from humans to humans can be written as

3 $${\beta }_{HH}={\beta }_{3}\frac{({k}_{1}{E}_{H}+{k}_{2}{I}_{H1}+{I}_{H2}+{k}_{3}{I}_{H3})}{{N}_{H}},$$

in which *m* is the number of female mosquitoes per person, *a* is biting rate of natural mosquitoes, *β*_1_ is transmission probability from natural mosquitoes to humans, *β*_2_ is transmission probability from humans to mosquitoes, *β*_3_ is transmission probability from humans to humans. *η*_*f*_ and *η*_*i*_ are the modification parameters, *i* = 1, 2, 3.

Based on the flow diagram in Fig. 2, we first formulate a transmission model for Zika virus without considering the control of vector mosquitoes:

4 $$\begin{array}{c}\{\begin{array}{ccc}\frac{d{S}_{H}}{dt} & = & -{\beta }_{fH}(1-{\varphi }_{H}){S}_{H}-{\beta }_{HH}{S}_{H}+p{\gamma }_{1}{I}_{H1},\\ \frac{d{E}_{H}}{dt} & = & {\beta }_{fH}(1-{\varphi }_{H}){S}_{H}+{\beta }_{HH}{S}_{H}-(1-z){\sigma }_{1}{E}_{H}-z{\sigma }_{2}{E}_{H},\\ \frac{d{I}_{H1}}{dt} & = & \theta (1-z){\sigma }_{1}{E}_{H}-{\gamma }_{1}{I}_{H1},\\ \frac{d{I}_{H2}}{dt} & = & z{\sigma }_{2}{E}_{H}+(1-p){\gamma }_{1}{I}_{H1}-{\gamma }_{2}{I}_{H2},\\ \frac{d{I}_{H3}}{dt} & = & (1-\theta )(1-z){\sigma }_{1}{E}_{H}-{\gamma }_{3}{I}_{H3},\\ \frac{d{R}_{H}}{dt} & = & {\gamma }_{2}{I}_{H2}+{\gamma }_{3}{I}_{H3},\\ \frac{d{A}_{f}}{dt} & = & {\rho }_{f}{F}_{f}(1-\frac{{A}_{f}}{K})-({\omega }_{f}+{\mu }_{fA}){A}_{f},\\ \frac{d{S}_{f}}{dt} & = & l{\omega }_{f}{A}_{f}-{\beta }_{Hf}{S}_{f}-{\mu }_{f}{S}_{f},\\ \frac{d{E}_{f}}{dt} & = & {\beta }_{Hf}{S}_{f}-{\sigma }_{f}{E}_{f}-{\mu }_{f}{E}_{f},\\ \frac{d{I}_{f}}{dt} & = & {\sigma }_{f}{E}_{f}-{\mu }_{f}{I}_{f},\end{array}\end{array}$$

where all the parameters are defined and summarized in Table 1.

During an edemic of the virus, the traditional insecticides are used to eliminate mosquitoes. But usually the effect is not satisfactory due to the insecticide resistance. Next, we establish new models by introducing bacterium Wolbachia into *Aedes aegypti* mosquitoes. Two models considering two different releasing strategies of Wolbachia-harboring mosquitoes will be established.

### Dynamical models with releasing of Wolbachia-harboring mosquitoes

During a mosquito season, the traditional insecticides are usually used to reduce the number of mosquitoes. Next, we will consider the novel vector control based on mosquito genetic modification by introducing bacterium Wolbachia into *Aedes aegypti* mosquitoes. We will extend model (4) to establish two new models considering two different strategies of releasing Wolbachia-harboring mosquitoes.

#### Model in simultaneously releasing Wolbachia-harboring female and male mosquitoes

To investigate the effect of releasing Wolbachia-harboring female and male mosquitoes on transmission of Zika, we assume that the quantities of Wolbachia-harboring female and male augmentation rate per week are Λ_*F*_ and (1 −* l*)Λ_*F*_/ *l*, respectively. Similarly to what we have in the model (4), we denote the total Wolbachia-harboring mosquitoes population at time *t* by *N*_*h*_, denote the population of aquatic stage by *A*_*h*_, we also classify them into classes of susceptible (*S*_*h*_), exposed (*E*_*h*_) and infectious (*I*_*h*_), then *N*_*h*_ = *A*_*h*_ + *F*_*h*_, where* F*_*h*_ = *S*_*h*_ + *E*_*h*_ + *I*_*h*_ is the total adult female Wolbachia-harboring mosquito population. Recall t﻿hat *M﻿*_*f*_ denotes the total adult Wolbachia-﻿free male mosquito population, similarly, we use *M*_*h*_ to denote the total adult male mosquito population, and in the rest of the paper we use the subscript *h* to rep﻿resent Wolbachia-harboring mosquitos. Then we have the sub-systems for human, Wolbachia-free mosquitoes and Wolbachia-harboring mosquitoes respectively as following:

For human population:

5 $$\begin{array}{c}\{\begin{array}{ccc}\frac{d{S}_{H}}{dt} & = & -{\beta }_{fH}(1-{\varphi }_{H}){S}_{H}-{\beta }_{hH}(1-{\varphi }_{H}){S}_{H}-{\beta }_{HH}{S}_{H}+p{\gamma }_{1}{I}_{H1},\\ \frac{d{E}_{H}}{dt} & = & {\beta }_{fH}(1-{\varphi }_{H}){S}_{H}+{\beta }_{hH}(1-{\varphi }_{H}){S}_{H}+{\beta }_{HH}{S}_{H}-(1-z){\sigma }_{1}{E}_{H}-z{\sigma }_{2}{E}_{H},\\ \frac{d{I}_{H1}}{dt} & = & \theta (1-z){\sigma }_{1}{E}_{H}-{\gamma }_{1}{I}_{H1},\\ \frac{d{I}_{H2}}{dt} & = & z{\sigma }_{2}{E}_{H}+(1-p){\gamma }_{1}{I}_{H1}-{\gamma }_{2}{I}_{H2},\\ \frac{d{I}_{H3}}{dt} & = & (1-\theta )(1-z){\sigma }_{1}{E}_{H}-{\gamma }_{3}{I}_{H3},\\ \frac{d{R}_{H}}{dt} & = & {\gamma }_{2}{I}_{H2}+{\gamma }_{3}{I}_{H3}.\end{array}\end{array}$$

For Wolbachia-free mosquitoes:

6 $$\begin{array}{c}\{\begin{array}{ccc}\frac{d{A}_{f}}{dt} & = & {\rho }_{f}{F}_{f}(1-\frac{\bar{q}{F}_{h}}{{F}_{f}+{F}_{h}})(1-\frac{{A}_{f}+{A}_{h}}{K})-({\omega }_{f}+{\mu }_{fA}){A}_{f},\\ \frac{d{S}_{f}}{dt} & = & l{\omega }_{f}{A}_{f}+l(1-\alpha ){\omega }_{h}{A}_{h}-{\beta }_{Hf}{S}_{f}-{\mu }_{f}{S}_{f},\\ \frac{d{E}_{f}}{dt} & = & {\beta }_{Hf}{S}_{f}-{\sigma }_{f}{E}_{f}-{\mu }_{f}{E}_{f},\\ \frac{d{I}_{f}}{dt} & = & {\sigma }_{f}{E}_{f}-{\mu }_{f}{I}_{f}.\end{array}\end{array}$$

For Wolbachia-harboring mosquitoes:

7 $$\begin{array}{c}\{\begin{array}{ccc}\frac{d{A}_{h}}{dt} & = & {\rho }_{h}{F}_{h}(1-\frac{{A}_{f}+{A}_{h}}{K})-({\omega }_{h}+{\mu }_{hA}){A}_{h},\\ \frac{d{S}_{h}}{dt} & = & {{\rm{\Lambda }}}_{F}+\alpha l{\omega }_{h}{A}_{h}-{\beta }_{Hh}{S}_{h}-{\mu }_{h}{S}_{h},\\ \frac{d{E}_{h}}{dt} & = & {\beta }_{Hh}{S}_{h}-{\sigma }_{h}{E}_{h}-{\mu }_{h}{E}_{h},\\ \frac{d{I}_{h}}{dt} & = & {\sigma }_{h}{E}_{h}-{\mu }_{h}{I}_{h}.\end{array}\end{array}$$

Due to the releasing of Wolbachia-harboring mosquitoes, some rates in model equations (5)–(7) are different from the model (4) in the absence of Wolbachia. In this model, a susceptible human also becomes exposed after being bitten by Wolbachia-harboring infectious or exposed mosquitoes at a rate of *β*_*hH*_ =* a*_1_ *β*_11_(*η*_*h*_ *E*_*h*_ + *I*_*h*_)/*N*_*H*_. Susceptible Wolbachia-harboring mosquitoes become exposed after biting exposed or infectious humans at a rate of *β*_*Hh*_  = *a*_1_ *β*_22_ *(η*_1_ *E*_*H*_ + *η*_2_ *I*_*H*__1_ *+ I*_*H*__2_+ *η*_3_ *I*_*H*__3_ *)/N*_*H*_. The definitions of parameters are given in Table 1.

In the mosquito population, the effects of cytoplasmic incompatibility (CI) are included. Wolbachia-free aquatic mosquitoes are produced after Wolbachia-free male and female mosquitoes mate and their reproduction is limited by a carrying capacity, *K*,

$${\rho }_{f}{F}_{f}(1-\frac{\bar{q}{M}_{h}}{{M}_{f}+{M}_{h}})(1-\frac{{A}_{f}+{A}_{h}}{K}),$$

in which $\bar{q}\in \mathrm{[0,}\,\mathrm{1]}$ is a probability of CI mechanism results in zygotic death when a Wolbachia-harboring adult mates with Wolbachia-free adult female (here $\bar{q}=0.9$ ^45, 57^).

Since we assume that the ratio of the size of male and female mosquitoes is 1 − *l*:*l*, than *M*_*f*_ = (1 −*l*)*F*_*f*_/*l*, *M*_*h*_  = (1 − *l*)*F*_*h*_/*l*, and the above equation is reduced to

$${\rho }_{f}{F}_{f}(1-\frac{\bar{q}{F}_{h}}{{F}_{f}+{F}_{h}})(1-\frac{{A}_{f}+{A}_{h}}{K}).$$

The Wolbachia-harboring aquatic stage at a proportion, *α*, matures into become adult Wolbachia-harboring mosquitoes, and a proportion, (1 − *α*), matures into become Wolbachia-free adult (here *α* = 0.9). For the remaining types of mosquitoes, the transformation route is similar to those in model (4) in the absence of wolbachia.

#### Model for only releasing Wolbachia-harboring male mosquitoes

Assuming that the quantities of Wolbachia-harboring male augmentation rate per week is Λ_*M*_, then we have the following two sets of models: ﻿For human population:

8 $$\begin{array}{c}\{\begin{array}{ccc}\frac{d{S}_{H}}{dt} & = & -{\beta }_{fH}(1-{\varphi }_{H}){S}_{H}-{\beta }_{HH}{S}_{H}+p{\gamma }_{1}{I}_{H1},\\ \frac{d{E}_{H}}{dt} & = & {\beta }_{fH}(1-{\varphi }_{H}){S}_{H}+{\beta }_{HH}{S}_{H}-(1-z){\sigma }_{1}{E}_{H}-z{\sigma }_{2}{E}_{H},\\ \frac{d{I}_{H1}}{dt} & = & \theta (1-z){\sigma }_{1}{E}_{H}-{\gamma }_{1}{I}_{H1},\\ \frac{d{I}_{H2}}{dt} & = & z{\sigma }_{2}{E}_{H}+(1-p){\gamma }_{1}{I}_{H1}-{\gamma }_{2}{I}_{H2},\\ \frac{d{I}_{H3}}{dt} & = & (1-\theta )(1-z){\sigma }_{1}{E}_{H}-{\gamma }_{3}{I}_{H3},\\ \frac{d{R}_{H}}{dt} & = & {\gamma }_{2}{I}_{H2}+{\gamma }_{3}{I}_{H3}.\end{array}\end{array}$$

For mosquitoes:

9 $$\begin{array}{c}\{\begin{array}{ccc}\frac{d{A}_{f}}{dt} & = & {\rho }_{f}{F}_{f}(1-\frac{\bar{q}{M}_{h}}{{M}_{f}+{M}_{h}})(1-\frac{{A}_{f}}{K})-({\omega }_{f}+{\mu }_{fA}){A}_{f},\\ \frac{d{S}_{f}}{dt} & = & l{\omega }_{f}{A}_{f}-{\beta }_{Hf}{S}_{f}-{\mu }_{f}{S}_{f},\\ \frac{d{E}_{f}}{dt} & = & {\beta }_{Hf}{S}_{f}-{\sigma }_{f}{E}_{f}-{\mu }_{f}{E}_{f},\\ \frac{d{I}_{f}}{dt} & = & {\sigma }_{f}{E}_{f}-{\mu }_{f}{I}_{f},\\ \frac{d{M}_{h}}{dt} & = & {{\rm{\Lambda }}}_{M}-{\mu }_{h}{M}_{h}.\end{array}\end{array}$$

In the above equations (8)(9), the CI effect leads to the death (or increased probability of death) of the offspring of Wolbachia-harboring males and Wolbachia-free females. Since these offspring are uninfected by Wolbachia, it effectively blocks Wolbachia-harboring female mosquitoes reproduction. So, Wolbachia-harboring female mosquitoes do not appear in the equations (8)(9). The involving parameters are also summarized in Table 1.

## Results

### Parameters estimation and basic reproduction number

Some parameters and their values are given in Table 1, while other parameters will be estimated by applying the model (4) to simulate the reported suspected cases of Zika infection in Brazil from February 6 to June 2, 2016.

We fit the model (4) using Markov-chain Monte Carlo (MCMC) simulations. Parameters *ϕ*_*H*_, *z*, *p*, *β*_3_, and *l* are estimated by fitting the model (4) with the limited amount of the reported data. We apply reported data on the new suspected human cases from the 5th week to 19th week in 2016 to fit the model (4). The relationship between the sizes of reporting new cases and that of modeling new suspected cases can be written as $qy(t)=\bar{y}(t)+\varepsilon$, where *y*(*t*) is theoretic new suspected case and $\bar{y}(t)$ is surveillance data in week *t*, *q* is report rate, *ε* follows a Gaussian distribution, that is $\varepsilon \sim N\mathrm{(0,\ }{\sigma }^{2})\,(\sigma =\mathrm{0.01)}$. *y*(*t*) = *I*_*H*11_(*t*) − *I*_*H*11_(*t* − 1), *t* = 6, 7, …, 19, with $qy\mathrm{(5)}=\bar{y}\mathrm{(5)}$, where *I*_*H*11_(*t*) is the accumulated suspected cases and its change with time is described as *dI*_*H11*_(*t*)/ *dt* = *θ(1 − z)σ*_1_ *E*_*H*_. To calculate the likelihood of observing a particular number of cases in week *t*, $\bar{y}(t)$, we assume the number of reported suspected new cases in week *t* followed a normal distribution. If we denote the vector of parameters as Θ = (*ϕ*_*H*_, *z*, *p*, *β*_3_, *l*), the maximum likelihood for data $Y={\{\bar{y}(t)\}}_{t=5}^{T}\mathrm{,\ (}T=\mathrm{19)}$ can be calculated as:

10 $$L(Y|{\rm{\Theta }})=\prod _{t=5}^{T}\frac{1}{\sqrt{2\pi }\sigma }exp(-\frac{{(\bar{y}(t)-qy(t))}^{2}}{{\sigma }^{2}}).$$

So the joint posterior distribution of the parameters is *P*(Θ|*Y*) ∝ *L*(*Y*|Θ)*P*(Θ).

The procedure of the MCMC method is carried out as in ref. 53. First, we assume each of Θ follows a non-information prior distributions and set initial value Θ_0_. We then generate the value of the modeling cases based on new parameters for calculating the posterior likelihood *P*(Θ^*^|*Y*) according to Eq. (4). For each iteration, new values of *Y* are generated from an adaptive proposed distribution *P*(Θ^*^|Θ). The new value of Θ can be calculated. All new values Θ^*^ and *Y* ^*^ will be accepted with probability

$$min(1,\,\frac{P({{\rm{\Theta }}}^{\ast }|Y)q({{\rm{\Theta }}|{\rm{\Theta }}}^{\ast })}{P({\rm{\Theta }}|Y)q({{\rm{\Theta }}}^{\ast }|{\rm{\Theta }})}),$$

where *q*(Θ^*^|Θ), the proposed density is chosen to be a multivariate normal distribution. After a number of iterations, we can then analyze the statistics of the model parameters and estimate their values.

**Figure 3:**
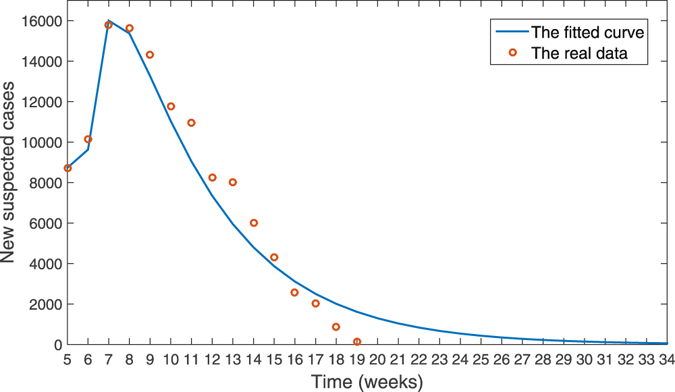
The fitting results of new suspected human cases from the 5th week to 19th week in 2016 and prediction result for the following 15 weeks. After the populations of both humans and mosquitoes are nondimensionalised, the initial human subpopulations are *S*_*H*0_ = 0.58, *S*_*E*0_ = *I*_*H*10_ = *I*_*H*20_ = *I*_*H*30_ = 0.0025. The initial mosquito subpopulations are *A*_*f*0_ = 0.6, *S*_*f*0_ = 0.45, *E*_*f*0_ = 0.15, *I*_*f*0_ = 0.4. Other parameters values are given in Table 1.

Based on the data of new reported suspected human cases in Brazil, and using MCMC method to fit our model (4), we estimate the sexual transmission rate as *β*_3_ = 0.04, which is close to 0.05 in literature^30^, and other unknown parameters estimated are given in Table 1. Figure 3 indicates that our model (4) provides reasonable fit to the reported Zika data from the 5th week to 19th week in 2016, in Brazil.

**Figure 4:**
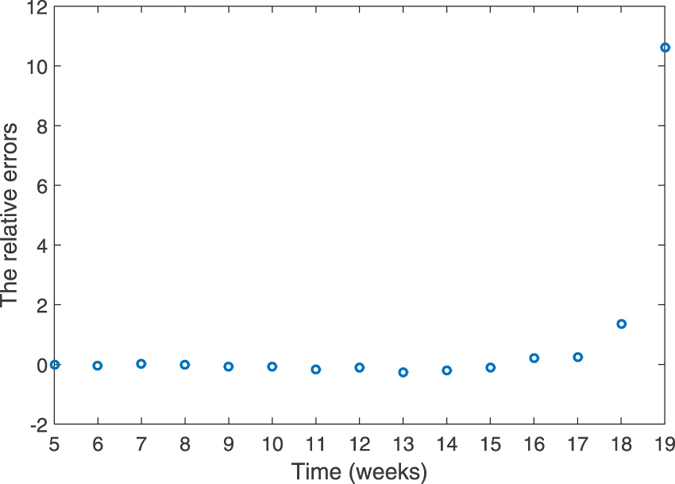
The relative error between theoretic and the real data.

By our simulation, we estimate that the number of the new suspected cases will reach a peak of 16000, which is close to the reported peak value of 15784. The peaking time estimated is in the 7th week which is same with the reported peaking time. The relative error between theoretic and the reported data is shown in Fig. 4. One can see from Fig. 4 that except for the 19th week, in general the relative errors are small and acceptable.

Through direct calculation, we obtain that the model (4) has a disease-free equilibrium ${E}_{0}=({S}_{H}^{0}\mathrm{,\ 0,\ 0,\ 0,\ 0,\ 0,\ }$ ${A}_{f}^{0}\mathrm{,\ }{S}_{f}^{0}\mathrm{,\ 0,\ 0)}$, in which ${S}_{H}^{0}=\mathrm{1,}$ ${A}_{f}^{0}=m[1-\frac{({\omega }_{f}+{\mu }_{fA}){\mu }_{f}}{l{\rho }_{f}{\omega }_{f}}],$ ${S}_{f}^{0}=\frac{l{\omega }_{f}{A}_{f}^{0}}{{\mu }_{f}}$. We calculate the basic reproduction number by using the next generation matrix. *R*_0_, the spectral radius of the next generation matrix, is given by

11 $${R}_{0}=\frac{{R}_{HH}+\sqrt{{R}_{HH}^{2}+4{R}_{HV}^{2}}}{2},$$

where *R*_*HH*_, partial reproduction numbers induced by mosquito vectorial transmission, is given by

12 $$\begin{array}{ccc}{R}_{HH} & = & [{k}_{1}+\frac{{k}_{2}\theta (1-z){\sigma }_{1}}{{\gamma }_{1}}+\frac{z{\sigma }_{2}+(1-p)\theta (1-z){\sigma }_{1}}{{\gamma }_{2}}+\frac{{k}_{3}(1-\theta )(1-z){\sigma }_{1}}{{\gamma }_{3}}]\frac{{\beta }_{3}{S}_{H}^{0}}{z{\sigma }_{2}+(1-z){\sigma }_{1}}.\end{array}$$

*R*_*HV*_, partial reproduction numbers induced by sexual transmission, is given by

13 $${R}_{HV}=\sqrt{\begin{array}{c}[{\eta }_{1}+\frac{{\eta }_{2}\theta (1-z){\sigma }_{1}}{{\gamma }_{1}}+\frac{z{\sigma }_{2}+(1-p)\theta (1-z){\sigma }_{1}}{{\gamma }_{2}}+\frac{{\eta }_{3}(1-\theta )(1-z){\sigma }_{1}}{{\gamma }_{3}}]\frac{{a}^{2}{\beta }_{1}{\beta }_{2}m(1-{\varphi }_{H})({\eta }_{f}{\mu }_{f}+{\sigma }_{f}){S}_{H}^{0}{S}_{f}^{0}}{{\mu }_{f}({\mu }_{f}+{\sigma }_{f})[z{\sigma }_{2}+(1-z){\sigma }_{1}]}.\end{array}}$$

We use above adaptive Metropolis-Hastings (M-H) algorithm to carry out the Markov-chain Monte Carlo procedure. Undetermined parameters are fitted. Then we estimate mean values *R*_0_ = 2.5020, where the term of *R*_0_ concerning the mosquito vectorial transmission is *R*_*HV*_ = 2.4750 and the sexual transmission is *R*_*HH*_ = 0.0538 which implies that two transmission routes play more important roles in the transmission of Zika in Brazil. As a result, *R*_*HV*_ is greater than *R*_*HH*_. This suggests that mosquito vectorial transmission is mainly factor to induce or sustain an outbreak. However, the effect of sexual transmission on the transmission of Zika cannot be neglected.

### Releasing Wolbachia-harboring mosquitoes and control of Zika

Based on model equations (5)–(9), the effects of novel control strategies on the transmission of Zika in Brazil are presented. Different strategies of releasing Wolbachia-harboring mosquitoes are compared.

**Figure 5:**
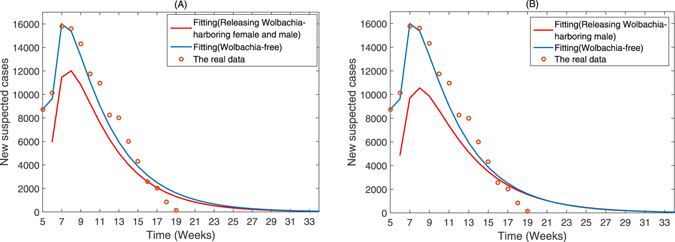
The effects of (**A**) simultaneously releasing Wolbachia-harboring female and male mosquitoes; (**B**) only releasing Wolbachia-harboring male mosquitoes. The initial human subpopulations are the same as Fig. 3. The initial mosquito subpopulations are *A*_*f*0_ = *A*_*h*0_ = 0.3, *S*_*f*0_ = *S*_*h*0_ = *E*_*f*0_ = *E*_*h*0_ = *I*_*h*0_ = 0.1,* M*_*h*0 ﻿_= ﻿0.3, *I*_*f*0_ = 0.15 for both control models. Releasing strength Λ_*M*_ = Λ_*F*_ = 0.3.

In this part, we consider the effect of releasing Wolbachia-harboring mosquitoes on the extent of the outbreak of Zika. There is no obvious effect in releasing initial phase, so we run the model until the infectious mosquito population decreases before the infected humans are introduced into the population on February 6, 2016. The contrast results of with and without control are shown in Fig. 5. The comparison in Fig. 5 indicates that if implemented for a long time, it can greatly reduce the magnitude of a outbreak. As shown in Fig. 5(A), after simultaneously releasing Wolbachia-harboring female and male mosquitoes, the peak decreases from 16000 to 12000 cases. It can also greatly reduce the magnitude from 16000 to 10560 cases of the outbreak when only releasing Wolbachia-harboring male mosquitoes from Fig. 5(B). From Fig. 5, we find that releasing Wolbachia-harboring mosquitoes is an effective methods to control the Zika in Brazil, and the effect is obvious.

**Figure 6:**
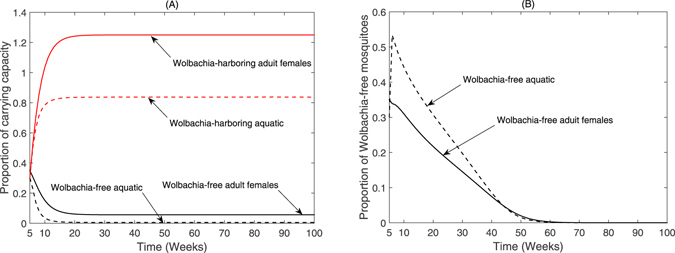
Numerical comparison with both aquatic and adult female mosquitoes. The change of ratio of mosquitoes after (**A**) simultaneously releasing Wolbachia-harboring female and male mosquitoes (Λ_*F*_ = 0.3), (**B**) releasing only Wolbachia-harboring male mosquitoes (Λ_*M*_ = 0.3). The solid curve represents adult mosquitoes and the dashed cure for aquatic mosquitoes.

Fig. 6 shows the density changes of natural and Wolbachia-harboring aquatic and adult mosquitoes over time under two different strategies of releasing Wolbachia-harboring mosquitoes. It follows from Fig. 6(A) that aquatic and adult Wolbachia-harboring mosquitoes increase and persist while aquatic and adult Wolbachia-free mosquitoes decrease with simultaneously releasing Wolbachia-harboring female and male mosquitoes over time. Fig. 6(B) gives that aquatic and adult Wolbachia-free mosquitoes decrease and until disappear along with only releasing Wolbachia-harboring male mosquitoes over time. This is because CI results in Wolbachia-free females produce an embryo upon mating with Wolbachia-harboring males, but the embryo is not viable and die. This feature suggest that continuing releasing Wolbachia-harboring male to cause CI, and thus suppress or even eradicate Wolbachia-free the effect^54, 55^ (see Fig. 6(B)). Moreover, Wolbachia-harboring females can reproduce successfully when mating with either Wolbachia-free or Wolbachia-harboring males. Whereas Wolbachia-free females can only reproduce successfully when mating with Wolbachia-free males. Since CI gives Wolbachia-harboring females a reproductive advantage, Wolbachia mosquitoes spread to the whole group, and it achieves population replacement effects^19, 56^ (see Fig. 6(A)). From Fig. 6, when strength of only releasing Wolbachia-harboring male mosquitoes is same that of simultaneously releasing Wolbachia-harboring female and male (Λ_*M*_ = Λ_*F*_ = 0.3), the corresponding reduced magnitude of Zika cases is more accordingly (see Fig. 5). We conclude that only releasing male Wolbachia mosquitoes will be a more effective option to control the spread of Zika virus. Next, we give further explanation about the effect of releasing only Wolbachia-harboring male on the size of Wolbachia-free population.

**Figure 7:**
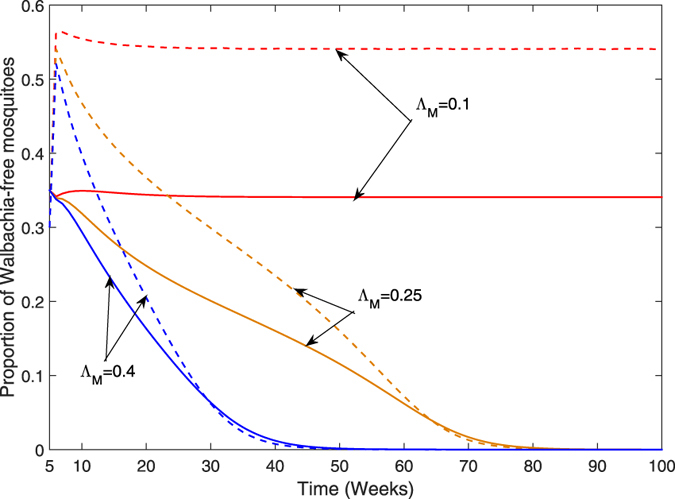
The effect of the strength Λ_*M*_ **of releasing only Wolbachia-harboring male on the size of Wolbachia-free population**. The solid curve represents adult mosquitoes and the dashed curve represents aquatic mosquitoes.

Strength of releasing Wolbachia-harboring male mosquitoes based on model (8)–(9) is one of the key factors since it affects vector population dynamics. We consider different strengths of releasing Wolbachia-harboring males, which are simulated by changing Λ_*M*_ from 0.1 to 0.4. From Fig. 7, as the strength (Λ_*M*_) of releasing increases, the size of Wolbachia-free population decreases. But, if we choose Λ_*M*_ = 0.1, it means that the release is much less effective. The results shown in Fig. 7 indicate that releasing Wolbachia-harboring male mosquitoes can reduce the size of Wolbachia-free population. Particularly, if the strength of releasing is increased Λ_*M*_ = 0.4, Wolbachia-free population will decrease and become extinguished.

L﻿et *﻿c* = *M*_*h*_/*M*_*f*_. We can calculat﻿e to obtain that *c* ≈ 5.06 if *Λ*_*M*﻿_ = 0.78. It means that if the strength of releasing Wolbachia-harboring male mosquitoes is set to ﻿0.78, this value can be converted to 5:1, the minimum ratio of releasing Wolbachia-harboring male mosquitoes over the wild male mosquitoes.

### Sensitivity

**Figure 8:**
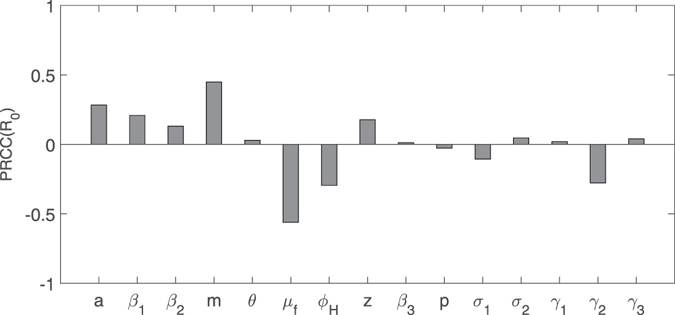
Partial rank correlation coefficients(PRCC) illustrating the dependence of basic reproduction number *R*_0_ **on each parameter.**

**Figure 9:**
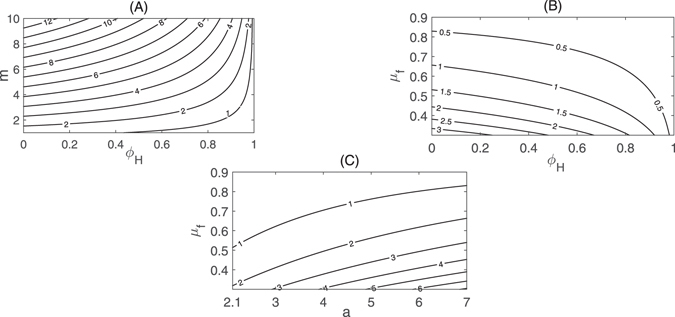
Contour plots of *R*_0_. Plot contours of *R*_0_ versus the proportion of person precaution *ϕ*_*H*_ and (**A**) the density of female mosquitoes per person *m*; (**B**) the death rate of adult Wolbachia-free mosquitos *μ*_*f*_; (**C**) Plot contours of *R*_0_ versus *α* and *μ*_*f*_. All other parameters values are as shown in Table 1.

In order to search critical parameters which make sense in Zika transmission, we can perform analysis of between critical parameters and the two outcome variables: the basic reproduction number *R*_0_ and accumulated suspected cases *I*_*H*11_ in model (4) by computing PRCC. It follows from Fig. 8 that *a*, *β*_1_, *m* and *z* are positively correlated with *R*_0_, while *μ*_*f*_, *ϕ*_*H*_ and *γ*_2_ are negatively correlated with *R*_0_. *R*_0_ is moderate or insensitive to variation of the rest parameters. The contour plots of Fig. 9 show the dependence of *R*_0_ on the mosquito biting rate *a*, the density of female mosquitoes per person *m*, the proportion of person precaution *ϕ*_*H*_ and the death rate of adult Wolbachia-free mosquitos *μ*_*f*_. Fig. 9(A) presents a combination of mosquito eradication and person precaution measures, decreasing *m* by 20% (while holding *ϕ*_*H*_ at the current level) can reduce *R*_0_ by 20.9%, and increasing *ϕ*_*H*_ by 20% (while holding *m* at the current level) can reduce *R*_0_ by 4.4%. Fig. 9(B) shows that *R*_0_ decreases from 2.5020 to 0.065 as *ϕ*_*H*_ increases to 1 or *μ*_*f*_ increases to 0.9. With baseline parameter values (listed in Table 1), the outbreak can be controlled when ${\mu }_{f}\, > \,\mathrm{0.83,\ }\forall a\in \mathrm{(2.1,}\,\mathrm{7)}$ (corresponding to *R*_0_ < 1) from Fig. 9(C). So the control strategies, decreasing *a* (or *m*) and increasing *μ*_*f*_ (or *ϕ*_*H*_), reduce the control *R*_0_.

**Figure 10:**
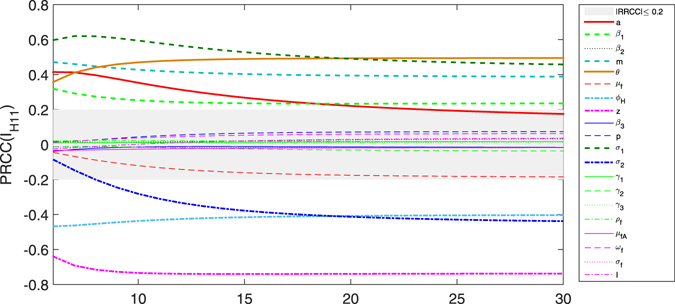
Plot of the PRCC over time of the model (4). The PRCC is calculated with respect to accumulated suspected cases *I*_*H*11_. The grey area indicates the region where the PRCC is not significantly different from zero (significance level 0.2), using 2000 samples.

To assess whether significance of one parameter occur over an entire time interval during the progression of the model dynamics, PRCC indexes for multiple time points and plotted versus time are calculated. Fig.  10 shows that the significance of the effect of parameters on the output *I*_*H*11_. For most of the time period, the most influential parameters are *a*, *β*_1_, *m*, *ϕ*_*H*_, *θ*, *z*, *σ*_1_ and *σ*_2_. In particular, *σ*_2_ becomes more and more correlated to the *I*_*H*11_ over time. *a* and *ϕ*_*H*_ become less and less correlated to the output over time; *m*, *θ* and *σ*_1_ are consistently significantly correlated to the output over time. For Fig. 10, the biting rate *a* decreases from moderate correlation to low correlation with the implementing measures of person precautions and mosquito eradication over epidemic outbreak. While it is worth noting that parameter *z*, associated with the proportion of *E*_*H*_ enter confirmed compartment, is consistently significantly negatively correlated to *I*_*H*11_ over time. The parameter *σ*_1_ determines the progression rate of humans from the exposed to suspected and asymptomatic infectious class. If this parameter is high, the exposed cases will quickly move to the suspected infectious class. So *σ*_1_ is consistently significantly correlated to *I*_*H*11_ over time. As the epidemic takes off, the cumulative numbers of suspected infectious individuals are determined more by the density of female mosquitoes per person *m*, the successful transmission probability *β*_1_, the proportion of symptomatic infection *θ* and the proportion of *E*_*H*_ enter confirmed compartment *z*.

## Conclusions and Discussions

### Conclusions without control

Based on the transmission mechanism of the virus and assessment of the limited data on the reported suspected cases, we establish a mathematical model without control measures for the transmission dynamics of Zika between humans and mosquitoes. Different from the previous works^28–30^, we divide the infected individuals into three classes: suspected case *I*_*H*1_(*t*), confirmed case *I*_*H*2_(*t*) and asymptomatic case *I*_*H*3_(*t*), which conform to the actual situations of Zika in Brazil. Using the model (4) to fit the limited data of suspected human cases of Zika in Brazil, good fitting results of model (4) are obtained. For the reemerging mosquito-borne flavivirus, there are no effective control measures due to many reasons, in this case the basic reproduction number *R*_0_ = 2.5020, which is close to 2.055 (CI: 0.523–6.300) in literature^30^, where the term of *R*_0_ concerning the sexual transmission is *R*_*HH*_ = 0.0538 and mosquito vectorial transmission is *R*_*HV*_ = 2.4750 which implies that mosquito vectorial transmission plays more important role in Zika transmission in Brazil while the effect of sexual transmission on the transmission of Zika cannot be neglected.

### Conclusions in terms of control

We estimate the basic reproduction number of model (4) *R*_0_ = 2.5020 of Zika. Based on the sensitivity discussions, we get the mosquito biting rate *a*, the density of female mosquitoes per person *m* and the proportion of person precautions *ϕ*_*H*_ play strongly influential roles on the *R*_0_ and accumulated suspected cases *I*_*H*11_. So the outbreak may be controlled if mosquitoes breeding sites are decreased and the person precaution is increased during the early stages of the disease outbreak. Therefore, we find that the novel biochemical control strategies, releasing Wolbachia-harboring mosquitoes, are considered to take center-stage in Brazil in this paper. By analyzing, we obtain simultaneously releasing Wolbachia-harboring female and male mosquitoes will achieve the effects of population replacement, only releasing Wolbachia-harboring male mosquitoes will suppress or even eradicate natural mosquitoes. By comparing different strategies of releasing Wolbachia- harboring mosquitoes, we conclude that only releasing male Wolbachia mosquitoes will be a more effective option to control the spread of Zika virus. Furthermore, through the previous analysis, the strength of releasing Wolbachia-harboring male mosquitoes is set to 0.78, this value can be converted to 5:1, the minimum ratio of releasing Wolbachia-harboring male mosquitoes over the wild male mosquitoes. It is a rather challenging task to decide the minimum ratio of “sterilized” mosquitoes over wild natural mosquitoes for the purpose of control. There have been some field and lab studies, we will keep it a future work to decide an optimal ratio when the related data is available.

### Discussions

Climate factors can influence various aspects of life cycle of vector mosquitos, including mating, reproduction, biting behavior and mortality. Since we only consider the duration from February to June 2016, the seasonal variation in transmission as a result of climate factor is not taken into account in the current paper. Spatial heterogeneity and climate factors are valuable research for Zika transmission and will be an important topic in our future research.

## Electronic supplementary material

Supplementary material

## References

[1] Foy BD (2011). Probable non-vector-borne transmission of Zika virus, Colorado, USA. Emerg Infect Dis.

[2] Marcondes CB, Ximenes MFF (2016). Zika virus in Brazil and the danger of infestation by *Aedes (Stegomyia)* mosquitoes. Revista da Sociedade Brasileira de Medicina Tropical.

[3] Bogoch II (2016). Anticipating the international spread of Zika virus from Brazil. Lancet.

[4] Hayes EB (2009). Zika virus outside Africa. Emerging Infectious Diseases.

[5] Schulerfaccini L (2016). Possible association between Zika virus infection and Microcephaly-Brazil, 2015. Mmwr Morbidity & Mortality Weekly Report.

[6] Dick GWA, Kitchen SF, Haddow AJ (1952). Zika virus (I) Isolations and serological specificity. Transactions of the Royal Society of Tropical Medicine and Hygiene.

[7] Duffy MR (2009). Zika virus outbreak on Yap Island, Federated States of Micronesia. New England Journal of Medicine.

[8] Hancock WT, Marfel M, Bel M (2014). Zika virus, French Polynesia, South Pacific, 2013. Emerging Infectious Diseases.

[9] Campos GS, Bandeira AC, Sardi SI (2015). Zika virus outbreak, Bahia, Brazil. Emerging Infectious Diseases.

[10] Roa M (2016). Zika virus outbreak: reproductive health and rights in Latin America. Lancet.

[11] Mccarthy, M. US health officials investigate sexually transmitted Zika virus infections. *Bmj* **352** (2015). 10.1136/bmj.i1180 26921165

[12] World Health Organization (WHO), WHO statement on the first meeting of the International Health Regulations (2005) Emergency Committee on Zika virus and observed increase in neurological disorders and neonatal malformations, February 1, 2016. http://www.who.int/mediacentre/news/statements/2016/1st-emergency-committee-zika/en/ (Accessed on February 26, 2016).

[13] Zanluca C (2015). First report of autochthonous transmission of Zika virus in Brazil. Memorias do Instituto Oswaldo Cruz.

[14] Hemingway J, Ranson H (2000). Insecticide resistance in insect vectors of human disease. Annual Review of Entomology.

[15] Qi RF, Zhang L, Chi CW (2008). Biological characteristics of dengue virus and potential targets for drug design. Acta Biochimica Et Biophysica Sinica.

[16] Kirsten H, Peter H, Peter S, Arndt T, Werren JH (2008). How many species are infected with Wolbachia?-A statistical analysis of current data. Fems Microbiology Letters.

[17] Dutra HL (2016). Wolbachia blocks currently circulating Zika virus isolates in Brazilian *Aedes aegypti* mosquitoes. Cell Host & Microbe.

[18] Yakob L, Walker T (2016). Zika virus outbreak in the Americas: the need for novel mosquito control methods. Lancet Global Health.

[19] Hoffmann AA (2011). Successful establishment of Wolbachia in *Aedes* populations to suppress dengue transmission. Nature.

[20] Aliota, M. T., Peinado, S. A., Velez, I. D. & Osorio, J. E. The *wMel* strain of Wolbachia reduces transmission of Zika virus by *Aedes aegypti*. *Plos Neglected Tropical Diseases* **6** (2016). 10.1371/journal.pntd.0004677 PMC4849757 27124663

[21] Mehta, N. Brazil releases Wolbachia infected mosquitoes to fight dengue. http://www.livemint.com/Consumer/ T8Ok070nJy1O4zlW5C93aP/Brazil-releases-Wolbachia-infected-mosquitoes-to-fight-dengu.html. (Accessed 26 Sepetemer 2014).

[22] Laven H (1951). Crossing experiments with Culex strains. Evolution.

[23] Yen JH, Barr AR (1971). New hypothesis of the cause of cytoplasmic incompatibility in Culex pipiens L. Nature.

[24] O’Neill SL, Giordano R, Colbert AM, Karr TL, Robertson HM (1992). 16SrRNA phylogenetic analysis of the bacterial endosymbionts associated with cytoplasmic incompatibility in insects. Proceedings of the National Academy of Sciences of the United States of America.

[25] Watson GS (1959). On the evolutionary importance of cytoplasmic sterility in mosquitoes. Evolution.

[26] Ndii MZ (2012). Modelling the introduction of Wolbachia into *Aedes aegypti* mosquitoes to reduce dengue transmission. Anziam Journal.

[27] Zhang X, Tang S, Cheke RA (2015). Birth-pulse models of Wolbachia-induced cytoplasmic incompatibility in mosquitoes for dengue virus control. Nonlinear Analysis: Real World Applications.

[28] Kucharski AJ (2016). Transmission dynamics of Zika virus in island populations: A modelling analysis of the 2013–14 French Polynesia outbreak. Plos Neglected Tropical Diseases.

[29] Funk S (2016). Comparative analysis of dengue and Zika outbreaks reveals differences by setting and virus. PLoS neglected tropical diseases.

[30] Gao, D. *et al*. Prevention and control of Zika fever as a mosquito-borne and sexually transmitted disease. *Scientific Reports* **6** (2016). 10.1038/srep28070 PMC4911567 27312324

[31] Majumder, M. S. *et al*. Utilizing nontraditional data sources for near real-time estimation of transmission dynamics during the 2015–2016 Colombian Zika virus disease outbreak. *Jmir Public Health & Surveillance* **2** (2016). 10.2196/publichealth.5814 PMC4909981 27251981

[32] Nishiura H (2016). A theoretical estimate of the risk of microcephaly during pregnancy with Zika virus infection. Epidemics.

[33] World Health Organization, Zika cases from the World Health Organization. http://www.who.int/emergencies/zika-virus/situation-report/en/ (Accessed 5 February 2016).

[34] Brazil Ministry of Health, Zika cases from the Brazil Ministry of Health. http://combateaedes.saude.gov.br/pt/situacao-epidemiologica#informes (Accessed 21 November 2015).

[35] World Health Organization, Case definitions of Zika virus. http://www.paho.org/hq/index.php?option=com_ content& view=article& id=11117 (Accessed 1 April 2016).

[36] Andraud M, Hens N, Marais C, Beutels P (2011). Dynamic epidemiological models for dengue transmission: a systematic review of structural approaches. Plos One.

[37] Turley AP, Moreira LA, O’Neill SL, Mcgraw EA (2009). Wolbachia infection reduces blood-feeding success in the dengue fever mosquito, *Aedes aegypti*. Plos Neglected Tropical Diseases.

[38] Lc DCM (2011). Modeling the dynamic transmission of dengue fever: investigating disease persistence. Plos Neglected Tropical Diseases.

[39] Briggs G (2010). The endosymbiotic bacterium Wolbachia induces resistance to dengue virus in *Aedes aegypti*. Plos Pathogens.

[40] Chikaki E, Ishikawa H (2009). A dengue transmission model in Thailand considering sequential infections with all four serotypes. Journal of Infection in Developing Countries.

[41] Bearcroft WGC (1956). Zika virus infection experimentally induced in a human volunteer. Transactions of the Royal Society of Tropical Medicine & Hygiene.

[42] Chowell G (2007). Estimation of the reproduction number of dengue fever from spatial epidemic data. Mathematical Biosciences.

[43] Gourinat AC, O’Connor O, Calvez E, Goarant C, Dupontrouzeyrol M (2015). Detection of Zika virus in urine. Emerging Infectious Diseases.

[44] Musso D (2015). Potential sexual transmission of Zika virus. Emerging Infectious Diseases.

[45] Walker T (2011). The wMel Wolbachia strain blocks dengue and invades caged *Aedes aegypti* populations. Nature.

[46] Yang HM, Macoris MLG, Galvani KC, Andrighetti MTM, Wanderley DMV (2009). Assessing the effects of temperature on the population of *Aedes aegypti*, the vector of dengue. Epidemiology & Infection.

[47] Yeap HL (2011). Dynamics of the “popcorn” Wolbachia infection in outbred *Aedes aegypti* informs prospects for mosquito vector control. Genetics.

[48] Eshita, Y. *et al*. Vector competence of Japanese mosquitoes for dengue and West Nile viruses. *Pesticide Chemistry* 217–225 (2007).

[49] Wilder Smith A, Foo W, Earnest A, Sremulanathan S, Paton NI (2004). Seroepidemiology of dengue in the adult population of Singapore. Tropical Medicine & International Health Tm & Ih.

[50] Yebakima A (2004). Genetic heterogeneity of the dengue vector *Aedes aegypti* in Martinique. Tropical Medicine & International Health.

[51] Manore CA, Hickmann KS, Xu S, Wearing HJ, Hyman JM (2014). Comparing dengue and chikungunya emergence and endemic transmission in *A. aegypti and A. albopictus*. Journal of Theoretical Biology.

[52] Pandey A, Mubayi A, Medlock J (2013). Comparing vector-host and sir models for dengue transmission. Mathematical Biosciences.

[53] Shi B, Tan Q, Zhou XN, Liu J (2015). Mining geographic variations of Plasmodium vivax for active surveillance: a case study in china. Malaria Journal.

[54] O’Connor L (2012). Open release of male mosquitoes infected with a Wolbachia biopesticide: Field performance and infection containment. Plos Negl Trop Dis.

[55] Sinkins SP (2004). Wolbachia and cytoplasmic incompatibility in mosquitoes. Insect Biochemistry & Molecular Biology.

[56] Xi Z, Khoo CC, Dobson SL (2005). Wolbachia establishment and invasion in an *Aedes aegypti* laboratory population. Science.

[57] Shi B, Tan Q, Zhou XN, Liu J (2015). Mining geographic variations of Plasmodium vivax for active surveillance: a case study in China. Malaria Journal.

