# Supplementary material for: Modeling the transmission and control of Zika in Brazil
Source: Sci Rep. 2017 Aug 10;7:7721. doi: 10.1038/s41598-017-07264-y (PMC5552773; doi:10.1038/s41598-017-07264-y)
Supplement: Supplementary file 1 — Supplementary material [file 41598_2017_7264_MOESM1_ESM.pdf]

Supplementary material

Modeling the transmission and control of Zika in Brazil

Liping Wang[1], **Hongyong Zhao**[1*], **Sergio Muniz Oliva**[2], **Huaiping Zhu**[3],

[1] Department of Mathematics, Nanjing University of Aeronautics and Astronautics, Nanjing 210016, P. R. China

[2] Departamento de Matemática Aplicada, Instituto de Matemática e Estatística, Universidade de São Paulo, Rua do Matão, 1010, Cidade Universitária, CEP 05508-090, São Paulo SP, Brazil

[3] Lamps and Department of Mathematics and Statistics, York University, Toronto, ON, M3J 1P3, Canada

[*] Corresponding author: hyzhao1967@126.com

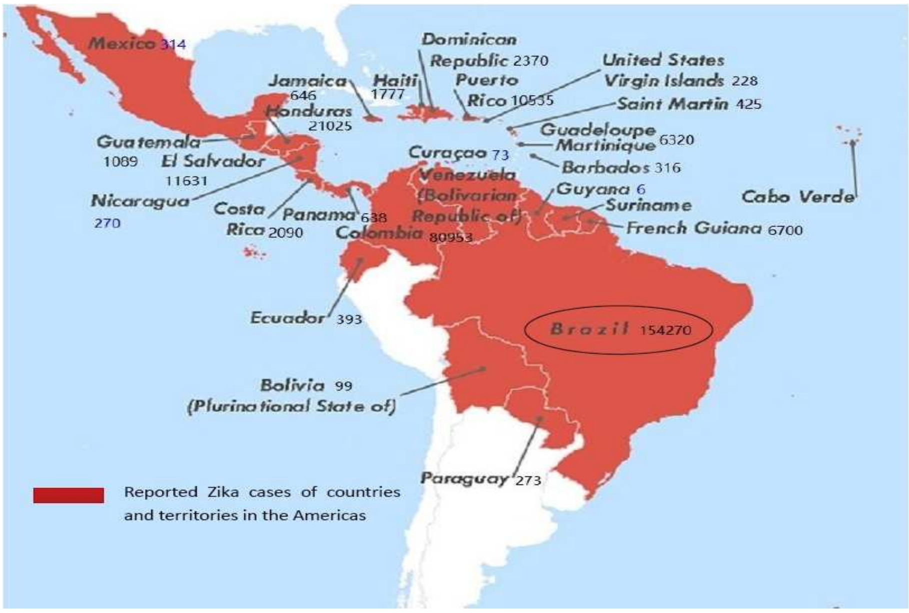

Supplementary Fig S1. Suspected and confirmed cumulative Zika cases were reported by countries and territories in the Americas, 2015-2016, up to June 2, 2016, in which 154270 suspected cumulative cases are notified for Brazil. Black Number represents suspected cumulative case, blue Number represents confirmed cumulative case. The map was made with the free software R (http://www.r-project.org) and data was from website(http://www.paho.org/hq/index.php?option=com_content&view=article&id=12390&Itemid=42090&lang=en) added by Microsoft Office Word 2007.
